# Prevention of Diet-Induced Obesity by Phytoecdysteroids 20-Hydroxyecdysone and Calonysterone—Unexpected Modulation of Androgen Balance in Normal and Obese Rats

**DOI:** 10.3390/nu18122023

**Published:** 2026-06-21

**Authors:** Alaa AM Osman, Dávid Laczkó, Máté Vágvölgyi, Noémi Tóth, Kata Kira Kemény, Péter Szatmári, Adrienn Seres-Bokor, Attila Hunyadi, Eszter Ducza

**Affiliations:** 1 Department of Pharmacodynamics and Biopharmacy, Faculty of Pharmacy, University of Szeged, Eötvös u. 6, H-6720 Szeged, Hungary; alaapharm27@gmail.com (A.A.O.); kemeny.kata.kira@szte.hu (K.K.K.); szapeti40@gmail.com (P.S.); seres-bokor.adrienn@szte.hu (A.S.-B.); 2 Institute of Pharmacognosy, Faculty of Pharmacy, University of Szeged, Eötvös u. 6, H-6720 Szeged, Hungary; david.laczko@cyclolab.hu (D.L.); vagvolgyi.mate@szte.hu (M.V.); crul-toth.noemi@szte.hu (N.T.); hunyadi.attila@szte.hu (A.H.); 3 HUN-REN-SZTE Biologically Active Natural Products Research Group, Eötvös u. 6, H-6720 Szeged, Hungary

**Keywords:** obesity, rat model, 20-hydroxyecdysone, calonysterone, androgen balance, skeletal muscle

## Abstract

Background: Calonysterone (CAL) is a natural derivative of 20-hydroxyecdysone (20E) with enhanced bioactivity on skeletal muscle cells in vitro, but its in vivo physiological actions remain less well characterized. This study aimed to compare the effects of 20E and CAL on metabolic, muscular, and endocrine parameters in normal and obese male rats, with a particular focus on androgen balance. Methods: Male rats were treated with 20E or CAL under normal (ND) or high-fat, high-sugar dietary (HFHSD) conditions for 12 weeks. Body weight, food intake, skeletal and androgen-sensitive muscle mass, and testicular weight were measured. Testicular expression of androgen receptor (*Ar*) and aromatase (*Cyp19a1*) mRNA was assessed by RT-PCR. ELISA was used to determine the plasma corticosterone, testosterone and ERβ level in testes. Results: 20E and CAL prevented HFHSD-induced weight gain and skeletal muscle atrophy. CAL uniquely preserved testicular and levator ani muscle mass in obese rats. CAL increased the expression of Cyp19a1 and ERβ in testicles. Decreased *Ar* mRNA expression was regulated in 20E- and CAL-treated obese animals. While 20E treatment significantly reduced plasma testosterone levels in the normal diet group compared to controls, both 20E and CAL interventions elicited significant reductions in the obese group relative to both the ND and HFHSD groups. HFHSD-induced increase in plasma corticosterone levels was normalized by 20E or CAL treatment. Conclusions: 20E and CAL exhibit beneficial metabolic and anabolic effects by preventing HFHSD-induced obesity and consequential muscle atrophy. CAL counteracts obesity-induced testicular atrophy in terms of tissue mass. Based on our results, we hypothesized that CAL enhances testicular aromatase levels, which may lead to increased compensatory androgen receptor mRNA expression and increased ERβ levels. These complex, not yet fully understood results underscore the need for caution in the use of phytoecdysteroids as dietary supplements.

## 1. Introduction

Obesity is a growing global health concern. According to the WHO estimates, approximately 59% of adults in the European Union are overweight or obese. Of this, around 17% of the EU adult population is clinically obese (with a BMI of 30 kg/m^2^ or higher) [1]. In men, obesity is often associated with reduced serum testosterone levels, disruptions in androgen balance, and an increased risk of metabolic syndrome, insulin resistance, and type 2 diabetes. These hormonal disturbances contribute to altered fat distribution, infertility, and deteriorating muscle mass and strength. Obesity is a paradoxical form of malnutrition and characterized with an excessive fat storage by lipid accumulation resulting from overconsumption of calories above the required amount of energy [2]. In addition to serving as an energy store, adipose tissue secretes numerous pro-inflammatory cytokines (e.g., tumor necrosis factor-alpha (TNF-α), interleukin-6 (IL-6), and IL-1β), hormones (e.g., leptin and adiponectin) and enzymes (e.g., cytochrome P450-dependent aromatase, CYP19A1) which interfere with the hypothalamic–pituitary–gonadal axis (HPA), disrupt the hormonal balance and contribute to the development of hypogonadism [3].

20-Hydroxyecdysone (20E), one of the most abundant phytoecdysteroids, is a naturally occurring polyhydroxylated steroid found in a variety of plant species. While initially identified for its role in insect development, phytoecdysteroids such as 20E are increasingly recognized for their pharmacological effects in mammals, including anabolic, anti-obesity, anti-inflammatory, antioxidant, and metabolic regulatory actions [4]. Notably, 20E has attracted significant attention in the fields of sports nutrition and functional food supplementation, promoted as a non-hormonal anabolic compound with low toxicity [5,6]. Its widespread use even led the World Anti-Doping Agency (WADA) to include 20E (syn. ecdysterone) on its monitoring list in 2020 as an anabolic agent in and out of competition. As of 2025, it is still being investigated for possible abuse in sports [7].

Consistent with their reported anabolic properties, ecdysteroids share some structural similarities with endogenous steroid hormones, prompting concerns about their potential interactions with the androgen, estrogen, and corticosteroid signaling pathways. Earlier in vitro and in vivo studies confirmed the non-hormonal anabolic activities of 20E and other ecdysteroids [5] in terms of stimulation of protein synthesis, increased weight of the whole body and internal organs, and improved skeletal muscle mass and fiber size [8,9]. Although 20E does not bind directly to the androgen receptor (*Ar*) [5], emerging evidence suggests it may exert some of its effects via estrogen receptor beta (ERβ) signaling [10,11]. ERβ is particularly relevant in skeletal muscle physiology, where its activation supports anabolic responses and muscle regeneration [12].

Despite preclinical evidence suggesting pharmacological activity of 20E, translation to humans remains contested. A major contributing factor is limited and variable bioavailability after oral administration, with circulating 20E and its metabolites exhibiting substantial inter-individual variability and uncertain pharmacokinetics [13], which complicates dose selection and the interpretation of efficacy. Moreover, the mechanism of action of 20E is not fully explored, with debates including the involvement of the Mas receptor and the role of GPCR/ERβ [3,14]. The direct binding of 20E to ERβ has not been consistently demonstrated, and results across experiments are not uniform [6,15]. Some studies emphasize non-nuclear ER-dependent signaling or membrane-associated receptor interactions that could cooperate with Mas receptor signaling.

Although the effects of 20E in mammals are well documented, considerably less is known about minor ecdysteroids. Calonysterone (CAL), a minor phytoecdysteroid and oxidized derivative of 20E, is regarded as a naturally occurring compound, having been isolated from kaladana seeds (*Ipomoea hederacea*) [16], *Vitex canescens* [17] and *Cyanotis arachnoidea* [18]. It is important to note, however, that CAL may also be formed artificially in *Cyanotis* extract during industrial plant processing [18]. Investigating the physiological effects of CAL is therefore relevant not only because it is a naturally occurring minor phytoecdysteroid but also because its relative abundance might increase during industrial preparation of *Cyanotis* extract, potentially leading to appreciable CAL exposure in consumers of ecdysteroid-containing food supplements [19]. In our previous study, both 20E and CAL similarly improved antioxidant capacity, normalized adipokine profiles, and increased DNA methylation in an obese rat model [20].

Previously, we demonstrated that 20E and its natural, oxidized derivative, calonysterone (CAL), improve antioxidant capacity, normalize adipokine profiles, and increase DNA methylation in an obese rat model [20]. Building on these results, we sought further to explore their effects on androgen balance and metabolic outcomes. CAL was synthesized in-house via base-catalyzed autoxidation of 20E and has previously been shown to activate Akt signaling more strongly in murine skeletal muscle cells than 20E [21], suggesting enhanced anabolic potential.

In the present study, we investigated the impact of 20E and CAL administration in male rats under normal and high-fat, high-sugar (HFHSD) dietary conditions, assuming that CAL alters endocrine function differently from 20E in vivo, particularly with regard to androgen axis regulation and testicular physiology. We aimed to assess their influence on body weight, skeletal and androgen-sensitive muscle mass, testicular mass, and hormonal parameters, including *Ar*, aromatase (*Cyp19a1*) mRNA, ERβ, testosterone and corticosterone levels. Given the increasing interest in ecdysteroids as natural performance enhancers, understanding their broader endocrine and metabolic effects is of both scientific and public health importance.

## 2. Materials and Methods

### 2.1. Preparation of Phytoecdysteroids

20E was commercially obtained from Shaanxi KingSci Biotechnology Co., Ltd. in Shanghai, China. According to the provider’s claim, it was extracted from the roots of *Cyanotis arachnoidea* and had an initial purity of 90%. To further improve purity, 20E was recrystallized twice. In each recrystallization step, 20E was dissolved in a minimal volume of hot methanol, and ethyl acetate was added gradually until slight turbidity persisted. A small amount of methanol was then added dropwise to restore a clear solution. Final solvent ratio was ethyl acetate and methanol at 2:1. The solution was allowed to cool slowly to room temperature and was left undisturbed for 24 h to promote crystallization. The resulting crystals were collected by filtration, washed with a cold ethyl acetate/methanol mixture, and dried under reduced pressure. A final purity of 96.9% was verified by high-performance liquid chromatography with diode array detection. This was then used to (i) treat the rats (20E treatment) and (ii) prepare CAL through base-catalyzed autoxidation, as previously reported [20,21]. Briefly, 20E (3 g) was dissolved in methanol (32 mL) and subsequently diluted with water (112 mL). An aqueous NaOH solution (2.4 g in 24 mL water) was added, and the reaction mixture was stirred at room temperature for 6 h. The mixture was then acidified with HCl and stirred overnight. Finally, the pH was adjusted to neutral using aqueous NaOH, and the solvent was removed under reduced pressure at 40 °C. CAL was purified by flash chromatography using a CombiFlash Rf+ system (Teledyne ISCO, Lincoln, NE, USA) equipped with RediSep C18 columns (Teledyne ISCO, Lincoln, NE, USA). Elution was performed with an aqueous methanol gradient of 25–50% MeOH (*v*/*v*). Fractions were pooled based on their TLC profiles, and those containing CAL were concentrated under reduced pressure at 40 °C, resulting in an isolated yield of 81%. HPLC–DAD analysis of the final purified CAL sample confirmed a chromatographic purity of 99.4% at 245 nm.

### 2.2. Housing and Handling of the Animals

All animal experiments in this study strictly complied with the ARRIVE guidelines and adhered to the European Communities Council Directive (2010/63/EU) and the Hungarian Act for the Protection of Animals in Research (Article 32 of Act XXVIII). The National Scientific Ethical Committee on Animal Experimentation approved all experiments involving animal subjects (registration number: IV/717/2023, approval date: 19 April 2023). All procedures were executed following relevant regulations and guidelines. Sprague–Dawley rats (INNOVO Ltd., Budapest, Hungary) were maintained in a controlled environment, at a relative humidity of 30–70% and a temperature of 22 ± 3 °C, and with a 12 h light and dark cycle.

Based on our previously known and accepted method, obesity was induced by nutritional intervention using a high-fat, high-sugar diet [20,22,23,24,25]. The animals were categorized into two distinct groups and provided with a specific diet, either a high-fat, high-sugar diet (HFHSD, comprising 28% fat: *saturated fatty acids*: arachidic acid (C-20:0)—120 mg/kg), palmitic acid (C-16:0)—6000 mg/kg, and stearic acid (C-18:0)—3240 mg/kg; *unsaturated fatty acids*: eicosanoic acid (C-20:1)—360 mg/kg alpha-linolenic acid (C-18:3)—360 mg/kg, linoleic acid (C-18:2)—68,400 mg/kg, and oleic acid (C-18:1)—32,400 mg/kg; 16% protein; and 56% carbohydrates: *disaccharides*: 176,505 mg/kg; *polysaccharides*: 356,027 mg/kg) (C1011, Altromin Spezialfutter GmbH & Co. KG, Lage, Germany, 3902 kcal/kg); or a normal diet (ND, comprising 14% fat: *saturated fatty acids*: arachidic acid (C-20:0)—148 mg/kg), palmitic acid (C-16:0)—5342 mg/kg, and stearic acid (C-18:0)—1615 mg/kg; *unsaturated fatty acids*: eicosanoic acid (C-20:1)—185 mg/kg alpha-linolenic acid (C-18:3)—3018 mg/kg, linoleic acid (C-18:2)—21,996 mg/kg, and oleic acid (C-18:1)—9287 mg/kg; 27% protein; and 59% carbohydrates: *disaccharides*: 54,151 mg/kg; *polysaccharides*: 350,300 mg/kg) (1314, Altromin Spezialfutter GmbH & Co. KG, Lage, Germany; 3339 kcal/kg) from the age of 6 weeks until the day of sacrifice, with tap water provided ad libitum. The animals’ weights and their food consumption were documented every week per cage of three rats. The caloric intake was calculated by multiplying the weight of the consumed food and the calorie value of the diet.

### 2.3. In Vivo Studies

Six-week-old Sprague–Dawley male rats (n = 36) were divided equally into six groups by a random number generator. Three groups of rats were fed with HFHSD to set as the obese groups, and three groups were fed the standard commercial rat chow (ND).

The rats were treated daily with a single dose of 10 mg/kg CAL or 20E based on the literature data. CAL and 20E were suspended in 0.25% mucilage methylcellulose (Sigma Aldrich, Budapest, Hungary) [20,26,27]. The rats were treated with CAL or 20E (10 mg/kg) in methylcellulose daily, by oral gavage [10], or they received no treatment. The following groups were set up: Group 1: high-fat, high-sugar diet (untreated, HFHSD), Group 2: HFHSD + 20E, Group 3: HFHSD + CAL, Group 4: normal diet control (untreated, ND), Group 5: ND + 20E, Group 6: ND + CAL.

Rat feeding and treatments continued for 12 weeks. Throughout the experiment, we measured both the amount of food consumed per cage and the body weight of each animal on a weekly basis. At the end of the study, the animals were sacrificed under deep isoflurane by blood collection and exsanguination through cardiac puncture (AErane liquid for inhalation, Baxter Hungary Ltd., Budapest, Hungary), with anesthesia using a Vaportec Isoflurane Vaporiser (Burtons Medical Equipment Ltd., Kent, UK) with a Fluovac Anesthetizing System (Harvard apparatus, Holliston, MA, USA); then, testicular tissues and the *levator ani, tibialis*, and *anterior soleus* muscles were collected for analysis.

**Figure 1:**
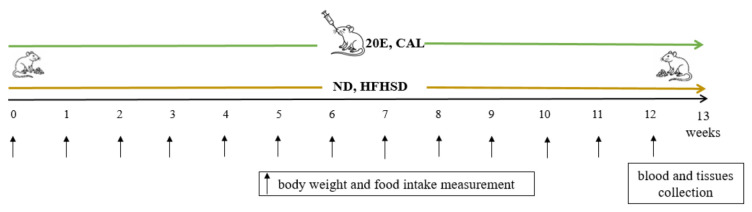
Animal experiment flowchart. Rat feeding and treatments continued for a duration of 12 weeks from the age of 6 weeks. HFHSD: high-fat, high-sugar diet; ND: normal diet; 20E: 20-hydroxyecdysone; CAL: calonysterone; ↑ first day of week.

For all experimental endpoints, the reported group size (n = 6 rats per group across the six experimental groups) remained constant, and all measurements were performed using the same cohort of animals. Biological replicates are defined as individual, independently treated animals, while technical replicates refer to laboratory assays performed in triplicate. Each tissue was collected by the same person, and weight measurements were performed by an assistant blinded to group allocation. The experimental design is illustrated in Figure 1.

### 2.4. RT-PCR Studies

*Tissue isolation.*

The testicles were collected and placed in RNAlater Solution (Sigma-Aldrich, Budapest, Hungary) and then stored at −80 °C until the extraction of total RNA.

*Total RNA preparation and real-time quantitative reverse transcription-PCR (RT-PCR).*

Total RNA preparation and RT-PCR were conducted in accordance with our previously published work [20,28]. The primers utilized included assay ID: Rn00560747_m1 for *Ar*, Rn00567222_m1 for aromatase (*Cyp19a1*) and Rn00667869_m1 for β-actin as endogenous control (Thermo Fisher Scientific, Budapest, Hungary).

### 2.5. ELISA Assays

Following the manufacturers’ recommendations, levels of estrogen beta (cat. no: ER0661, FineTest, Wuhan, China), testosterone (cat. no: ER1462, FineTest, Wuhan, China) and corticosterone (cat. no: ER0859, FineTest, Wuhan, China) were measured by rat ELISA kits. Plasma was used with a 2-fold dilution, and samples were run in duplicate.

### 2.6. Statistical Analysis

Statistical analysis was performed using the Prism 9.0 software (Graphpad Software Inc., San Diego, CA, USA). The normal distribution was tested for each group separately by the Shapiro–Wilk test. Two-way analysis of variance (ANOVA) was used to evaluate the effects of treatments and diet types on the parameters investigated. Tukey’s post hoc test was applied to the analyses. Each parameter was measured in three technical replicates, and mean values were used for further analysis. Each data point is presented as the mean ± standard error of the mean (SEM) of three independent measurements, and statistical significance was accepted at *p* < 0.05.

## 3. Results

### 3.1. Metabolic Effects of 20E and CAL in Rats

**Figure 2:**
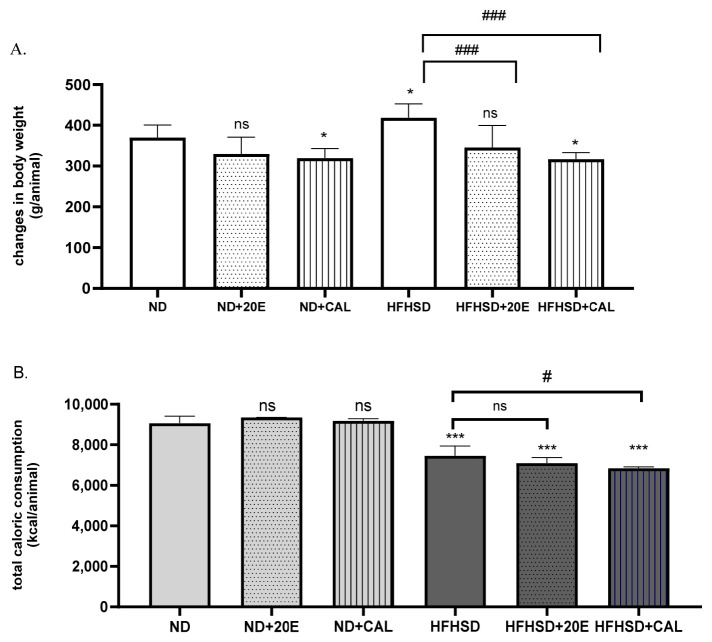
Changes in body weight in different groups until the end of the study (**A**) and caloric consumption (**B**) during the study. ND: normal diet; HFHSD: high-fat, high-sugar diet; 20E: 20-hydroxyecdysone; CAL: calonysterone treatments; ns: *p* > 0.05; *: *p* < 0.05; ***: *p* < 0.001 compared to the normal diet group (ND). #: *p* < 0.05; ###: *p* < 0.001 compared to the HFHSD group.

The changes in body weight show a significant increase in the HFHSD group compared with the control group (ND). The daily administration of ecdysteroid (20E or CAL) treatments for 12 weeks prevented the weight-gain effect of the HFHSD. Interestingly, CAL significantly decreased the body weight in the normal diet control group (Figure 2A).

Despite weight gain, the caloric intake was significantly lower in the HFHSD group compared to ND (Figure 2B).

### 3.2. Effects of 20E and CAL on Muscle and Reproductive Tissue Morphology

**Figure 3:**
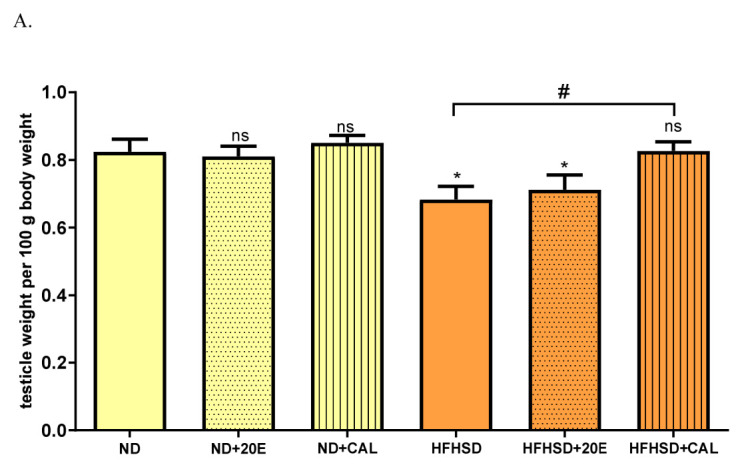

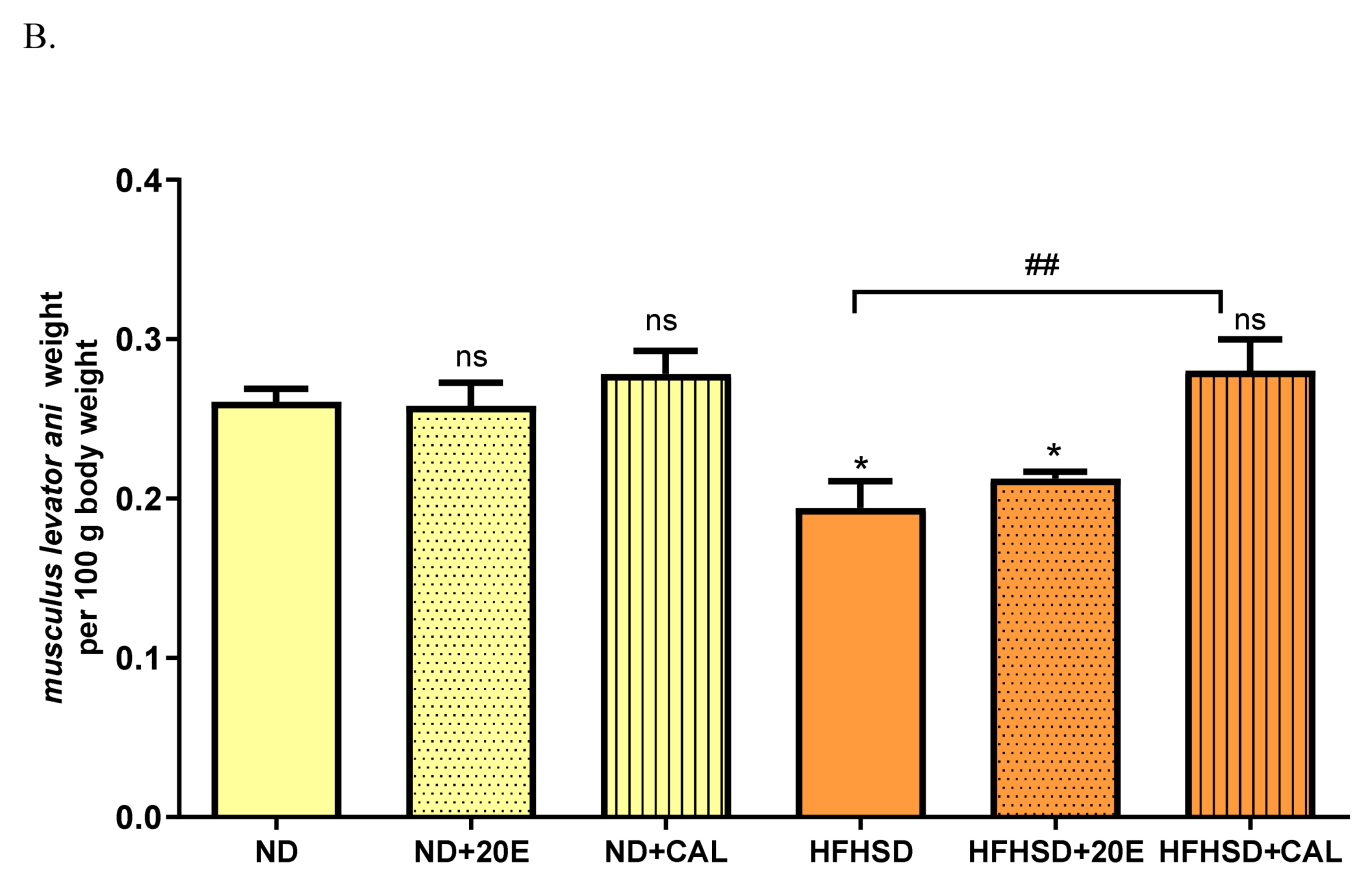
Changes in testicle (**A**) and musculus levator ani (**B**) weight per 100 g body weight. ND: normal diet; HFHSD: high-fat, high-sugar diet; 20E: 20-hydroxyecdysone; CAL: calonysterone treatments; ns: *p* > 0.05; *: *p* < 0.05 compared to the normal diet (ND). #: *p* < 0.05; ##: *p* < 0.01 compared to the HFHSD group.

The HFHSD resulted in reduced testicular (Figure 3A) and *musculus levator ani* mass (Figure 3B), which the 20E treatment could not prevent. In contrast, CAL administration normalized testicular and *musculus levator ani* weights. The weight of the testicle increased by 21.11% and that of the musculus levator ani by 43.94% compared to the untreated HFHSD group. In the ND groups, we did not observe significant changes upon ecdysteroid treatment.

**Figure 4:**
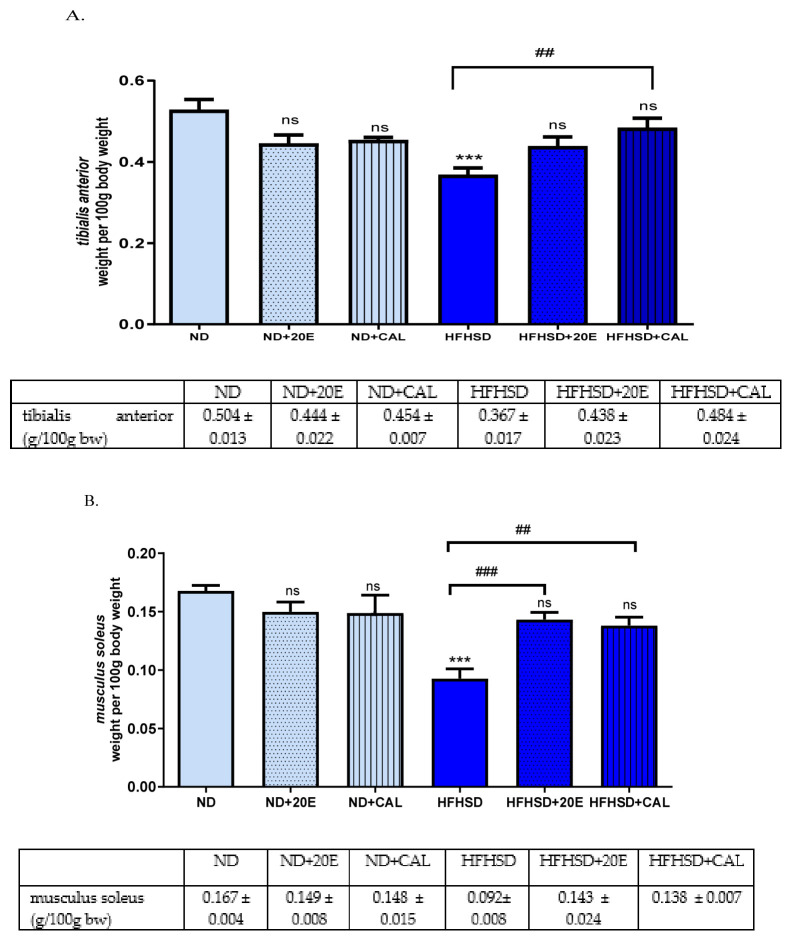
Changes in rat tibialis anterior (**A**) and musculus soleus (**B**) weight per 100 g body weight. ND: normal diet; HFHSD: high-fat, high-sugar diet; 20E: 20-hydroxyecdysone; CAL: calonysterone treatments; ns: *p* > 0.05; ***: *p* < 0.001 compared to the control (ND). ##: *p* < 0.01; ###: *p* < 0.001 compared to the HFHSD group.

The skeletal muscle tissues (*tibialis anterior*, *musculus soleus*) were shrunk by the HFHSD. Treatment with 20E or CAL protected the animals in the HFHSD groups from this effect of the diet. No significant effect on muscle mass was observed in the ND groups (Figure 4).

### 3.3. Effect of 20E and CAL on Endocrine and Molecular Signaling

#### 3.3.1. Alteration in Aromatase (Cyp19A1) and Ar mRNA Expression in Testicles

We examine changes in aromatase mRNA expression in the testis of the ND group and found that it increases significantly after CAL treatment.

Compared with the ND and HFHSD groups, mRNA expression in the untreated HFHSD and CAL-treated HFHSD groups was significantly higher than in the untreated, normal diet group.

**Figure 5:**
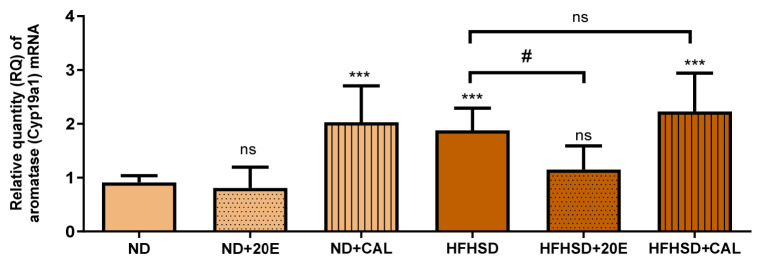
Alterations in aromatase mRNA expression in the rat testicular tissues. ND: control (standard diet); HFHSD: high-fat, high-sugar diet; 20E: 20-hydroxyecdysone; CAL: calonysterone treatments; ns: *p* > 0.05; ***: *p* < 0.001 compared to the normal diet control (ND). #: *p* < 0.05 compared to the HFHSD group.

In the obese group, 20E treatment decreased, whereas CAL treatment did not significantly alter aromatase expression compared with untreated HFHSD (Figure 5).

The mRNA expression pattern of change was similar in the HFHSD and ND groups regarding *Ar* mRNS expression.

In the ND groups, the *Ar* mRNA expression was higher after treatment, but this difference was significant only in the CAL-treated group.

**Figure 6:**
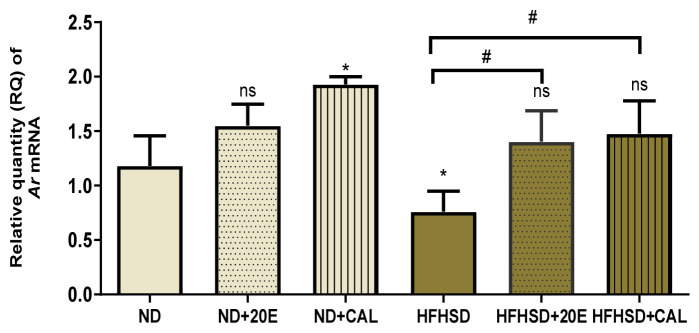
Alterations in *Ar* mRNA expression in testicular tissues. ND: normal diet; HFHSD: high-fat, high-sugar diet; 20E: 20-hydroxyecdysone; CAL: calonysterone treatments; ns: *p* > 0.05; *: *p* < 0.05; compared to the ND. #: *p* < 0.05; compared to the HFHSD group.

When comparing the untreated HFHSD and ND groups, a significant decrease in *Ar* mRNA expression was observed in the HFHSD group, according to the literature data. In the HFHSD group, the treatments of 20E and CAL significantly increased *Ar* mRNA expression in testicular tissues compared with non-treated HFHSD (Figure 6).

#### 3.3.2. Alteration in Plasma Testosterone Level

**Figure 7:**
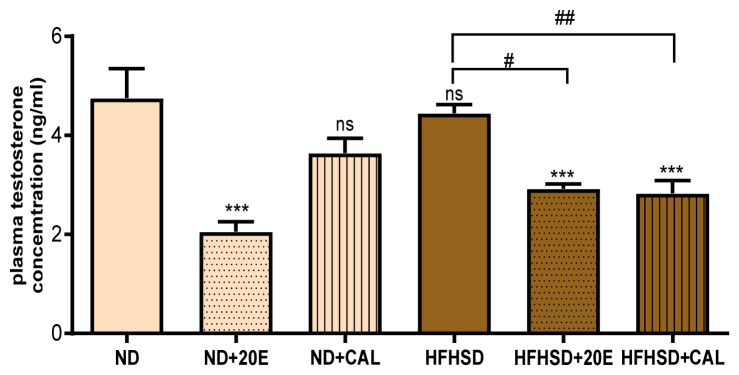
Alterations in testosterone level in the rat plasma. ND: control (normal diet); HFHSD: high fat high sugar diet; 20E: 20-hydroxyecdysone; CAL: calonysterone treatments; ns: *p* > 0.05; ***: *p* < 0.001 compared to the normal diet (ND). #: *p* < 0.05; ##: *p* < 0.01 compared to the HFHSD group.

Plasma testosterone levels were inversely correlated with *Ar* mRNA expression in both the normal and HFHS diet groups. In the normal diet group, the decrease after treatment was significant by 20E compared to the ND group. Comparing the untreated ND and untreated HFHSD group, there was no difference. In the obese group, 20E and CAL treatments significantly decreased plasma testosterone concentration compared with ND and HFHSD (Figure 7).

#### 3.3.3. Alterations in Plasma ERβ and Corticosterone Level

CAL effects differ significantly between the normal diet and HFHSD conditions in the changes in ERβ and corticosterone concentration.

**Figure 8:**
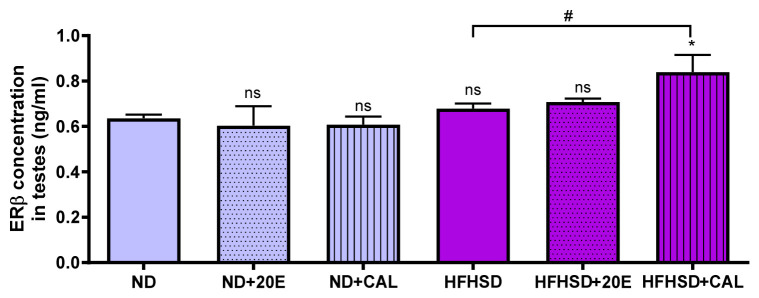
Changes in ERβ concentration in rat testes in different diet groups. ND: normal diet; HFHSD: high-fat, high-sugar diet; 20E: 20-hydroxyecdysone; CAL: calonysterone treatments; ns: *p* > 0.05; *: *p* < 0.05; compared to the control (ND). #: *p* < 0.05; compared to the HFHSD group.

ERβ overexpression was observed after CAL treatment in the HFHSD group compared to the untreated ND and HFHSD groups (Figure 8).

**Figure 9:**
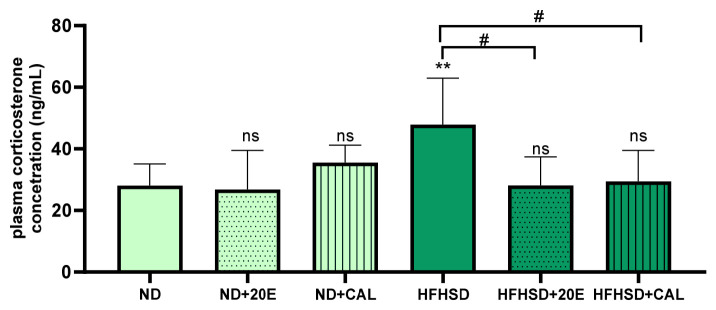
Changes in plasma corticosterone levels in rats in different diet groups. ND: normal diet; HFHSD: high-fat, high-sugar diet; 20E: 20-hydroxyecdysone; CAL: calonysterone treatments; ns: *p* > 0.05; **: *p* < 0.01; compared to the normal diet (ND). #: *p* < 0.05; compared to the HFHSD group.

Plasma corticosterone level was not altered by ecdysteroid treatment under ND conditions. In contrast, a significant increase in corticosterone concentration was observed in obese rats, which was normalized by treatment with 20E or CAL (Figure 9).

### 3.4. Diet and 20E and CAL Treatment Interaction

A significant diet and CAL treatment interaction was observed in changes in ERβ concentration (*p* = 0.0286, *), tibialis anterior (*p* = 0.0003, ***), *musculus soleus* (*p* = 0.0155, *), *m. levator ani* (*p* = 0.0323, *), testicular weight (*p* = 0.0361, *), body weight (*p* = 0.0126,*), and total caloric consumption (*p* < 0.0001, ***), indicating that the effect of CAL on these parameters depended on dietary condition. After 20E treatment, there was an interaction in changes of the *musculus soleus* (*p* = 0.0015, **) and total caloric consumption (*p* = 0.0004, ***), indicating that the effect of 20E on these parameters depended on dietary conditions.

## 4. Discussion

Plant-based diets are increasingly recognized not only for their role in promoting environmental sustainability but also for their inclusion of foods rich in bioactive phytochemicals, including phytoecdysteroids. Among these, 20E has received particular attention due to its reported anabolic, antioxidant, anti-obesity, and further potential therapeutic effects in chronic conditions such as fatigue, cardiovascular diseases, and neurodegenerative disorders [3,14]. Its widespread availability in dietary supplements, especially those targeting athletes, has led to increased regulatory attention, with the World Anti-Doping Agency (WADA) adding 20E to its monitoring program in 2020 [7]. Some studies suggest that 20E may signal via a G-protein-coupled membrane receptor [14], possibly involving a cross-talk with the Mas receptor of the renin–angiotensin system, leading to muscle hypertrophy [3]. Nevertheless, 20E is classified as a non-classical anabolic agent, lacking direct binding affinity to the androgen receptor (AR) [5], and emerging evidence suggests that its effects may be mediated through ERβ directly or indirectly rather than traditional androgenic pathways [15]. Because of this, it may as well be considered as a functional phytoestrogen.

Beyond their reproductive effects, phytoestrogens are increasingly being studied for their metabolic regulatory roles, including the potential to counteract obesity and its comorbidities. In males, obesity is commonly associated with hypogonadism, characterized by decreased total and free testosterone, elevated estrogen, and hypothalamic–pituitary–adrenal axis (HPA) overstimulation, leading to increased corticosteroid levels [29] and altered hypothalamic–pituitary–gonadal (HPG) axis function [30]. Contributing factors include oxidative stress, disrupted adipokine signaling, increased leptin and pro-inflammatory cytokines, reduced adiponectin, and enhanced aromatase activity in adipose tissue [31].

In our experimental model, we administered 20E and its oxidized derivative, CAL, to male rats under both standard and HFHSD conditions. We have previously reported that both compounds prevent HFHSD-induced weight gain, normalize adiponectin and leptin levels, and reduce circulating IL-6 and oxidative stress marker levels [20]. All these physiological effects are consistent with phytoestrogenic activity [32]. Weight reduction induced by 20E has been reported in rodent models [26,33]. We observed this effect with changes in food caloric consumption. This suggests that the anti-obesity actions of these compounds are likely mediated by modulation of metabolic pathways. For 20E, previous studies have shown enhancement of lipophagy [26], improvement in glucose tolerance and insulin sensitivity, suppression of adipogenesis [33,34], and inhibition of hepatic gluconeogenesis [35]. The current findings suggest that CAL may share similar mechanisms of action. It is worth noting that CAL also reduced the body weight of animals on a normal diet by the end of the experiment compared to 20E.

Obesity is closely linked to declining male fertility in both humans and rodent models, with high-fat diets and increasing BMI associated with reduced testosterone levels, sperm count and motility, and heightened oxidative stress and inflammation, i.e., factors that elevate the risk of oligozoospermia and azoospermia [36]. In the current study, the levator ani muscle and the testes showed differential responses to treatments: CAL, but not 20E, effectively preserved their mass along with testicular weight. Presumably, this phenomenon may be explained by different mechanisms of action resulting from structural differences among ecdysteroids. It can be stated that the effect profiles of 20E and CAL are different, the details of which require further studies, for example, with regard to ERβ selectivity or to alternative non-AR-mediated pathways (e.g., activation of the PI3K/Akt pathway or mTOR signaling).

Significant changes in body weight, total caloric consumption, *musculus levator ani* and testicular weight indicated that the effect of CAL on these parameters depended on the dietary condition. Similarly, the 20E treatment significantly altered total caloric consumption, demonstrating that the impact of 20E on these variables was likewise dependent on the dietary condition.

Skeletal muscle, which is also highly dependent on androgens for growth [37], is negatively affected by obesity-related testosterone decline and insulin resistance [38,39], as demonstrated in our studies. In our study, both ecdysteroids mitigated muscle atrophy in the tibialis anterior and soleus, which effect is consistent with their anabolic activity [5,6,8]. According to a recent phase 2b clinical study conducted with 20E, this compound may be used therapeutically in diseases associated with, e.g., age-related muscle wasting (sarcopenia) in the future [40]. The key to explaining the divergent effects across the testis, levator ani, and striated muscle tissue likely resides in the tissue-specific receptor distribution of ERβ [41,42]. Our results indicate that CAL’s impact on the two investigated skeletal muscles and 20E’s effect on the *musculus soleus* were modulated by dietary condition.

In rat testes, aromatase (the product of the *Cyp19a1* gene) plays a crucial role in maintaining the local androgen–estrogen balance, which is essential for normal spermatogenesis. Locally synthesized estrogens, mediated by aromatase, act through ERβ to regulate the survival and differentiation of germ cells and to modulate the steroidogenic capacity of Leydig cells. Chronic inflammation associated with obesity, and inflammatory factors (e.g., IL-6 and TNF-α) increase the expression of the *Cyp19a1* gene encoding the aromatase enzyme in testicular tissues [43,44]. This can also be observed in our results on obesity. 20E normalized the increased aromatase expression caused by obesity, while CAL did not, and it even increased it in the ND group.

In this study on aromatase mRNA expression, we report gene expression levels and did not examine the protein expression of androgen and estrogen signaling. With these limitations, we assume that this may be explained by the fact that obesity increases inflammatory cytokines (e.g., TNF-α), which continuously stimulate aromatase. Based on our previous studies, 20E may act as an anti-inflammatory stimulus and therefore “silence” this expression [20]. The effect of CAL on increasing aromatase expression in the testis is surprising, which may be part of a “feedback” mechanism. Furthermore, the aromatase gene is known to have tissue-specific promoters. It is a tempting hypothesis that CAL affects signaling pathways that directly or indirectly (via the PPAR system) stimulate the function of the testis-specific PII promoter [45]. Further studies are needed to confirm or refute this.

In obesity, AR activity is significantly reduced in key tissues, contributing to hormonal imbalance and metabolic dysfunction. The reason for this phenomenon is that the chronic inflammation associated with obesity (e.g., increased IL-6 and TNF-α levels) inhibits AR expression in the hypothalamus and testes [46]. 20E and CAL normalized the HFHSD-caused low AR expression in the testicular tissues. However, CAL also increased AR expression in the ND group, which may be a cause for concern. This may be explained by several suggested hypotheses. For example, an increase in the density of androgen receptors in the testis, or in other target organs, is usually an adaptive response of the body, aimed at normalizing hormonal efficiency. This allows for a stronger biological response even at lower hormone levels. This effect may explain the possible effect of CAL on the testicles. In addition to the increased AR expression, we also detected increased aromatase expression in the testis in the case of CAL. A tight, self-regulating feedback mechanism exists between the increase in AR density and the activity of the aromatase enzyme [47]. The product of aromatization, estradiol, may paradoxically influence AR expression. In certain tissues, an increase in estrogen levels signals the cell to increase the density of androgen receptors, thus maintaining the androgen/estrogen ratio. Our results may also be presumably explained by the fact that aromatase and androgen receptor gene expression are often regulated by the same signaling pathways. When a substance stimulates cellular metabolism in the testis (for example, in Leydig cells), it is often accompanied by an increase in the activity of both systems [48].

In our study, we found a significant decrease in plasma testosterone levels in both the ND and HFHSD groups. It is known from the literature data that the extent of the effect of ecdysteroids on plasma testosterone levels depends on dose, bioavailability, individual microbiome composition and genetic factors, making it difficult to draw uniform conclusions about the overall effect on plasma testosterone levels [49,50]. In the context of polycystic ovarian syndrome, phytoestrogens may exhibit effects that attenuate hyperandrogenism or improve insulin sensitivity. Some studies have suggested that phytoestrogens reduce total and free testosterone and may increase sex hormone binding proteins, which may lead to lower free testosterone levels, but other studies have not found significant changes [51]. We hypothesize that this decrease in testosterone levels is compensated for by the increase in androgen receptor mRNA expression observed in our studies.

It was previously suggested that the effects of 20E are at least partially mediated through ERβ [9]. Interestingly, unlike 20E, CAL increased ERβ expression in obese testicular tissue, thus confirming its different hormonal effects. The reasons for this can be determined by further studies, but we measured higher aromatase expression, which may have increased aromatase activity in CAL, leading to local estrogen excess. Estrogen receptors (especially ERβ) often regulate themselves in an autocrine manner; thus, higher estrogen levels may increase receptor synthesis through positive feedback [52].

The tissue-specific effects observed in the present study are consistent with findings reported for other plant-derived compounds. The soy isoflavone genistein has been shown to act as a tissue-selective androgen receptor modulator. In intact male mice, genistein acted as an androgen antagonist in the testes, prostate, and brain, whereas in castrated males, it acted as an androgen receptor agonist in the prostate and brain. In other androgen-sensitive tissues, such as skeletal muscle, however, it did not exhibit any antiandrogenic effects [53]. Overall, these observations support the idea that plant-derived bioactive compounds may exert selective effects on the male reproductive tract.

Furthermore, both compounds normalized the high corticosterone concentrations in HFHSD, an effect associated with reduced low-grade inflammation and muscle catabolism in obesity. This glucocorticoid-lowering effect aligns with previous observations of 20E and CAL’s anti-inflammatory actions and could contribute significantly to the maintenance of muscle mass [54]. Our earlier findings of reduced IL-6, increased SOD and catalase, and enhanced DNA methylation suggest a broader anti-inflammatory and epigenetic impact [20]. Similar to our result, in a study, Parr et al. also showed that a reduction in serum corticosterone levels accompanies the anabolic activity of 20E [9], which might also be explained by the inhibition of 11-β-hydroxysteroid dehydrogenase 1 enzyme expression reported for 20E [55].

It is important to consider the timing of exposure in our study. The rats in our study started being treated during a critical window of pre- and peripubertal development, a period when the endocrine system is highly plastic and vulnerable to disruption [56]. Previous animal studies have shown that high-dose phytoestrogens administered during early life stages can impair sexual development, reduce testosterone, and affect fertility parameters [57,58]. While we did not observe overt feminization or structural abnormalities in reproductive organs, the observed hormonal shifts and receptor expression changes raise concerns about long-term reproductive consequences.

Given the lack of global statistical corrections for multiple comparisons, individual significant differences should be interpreted with caution, as some findings may represent potential false positives. Therefore, our results should be viewed as an integrated pattern of biological changes and trends across the experimental groups rather than as independent, definitive, statistically confirmed effects.

## 5. Limitations

Limitations of our animal study investigating the effects of CAL and 20E include that the results are based on rats, are limited to male subjects, and do not clarify the safety of long-term chronic administration to other tissues. Our study does not detail the effects of different doses and the differences in bioavailability between rodents and humans. Our current results do not yet provide a precise explanation for the exact causes of each change because protein expression (AR, ERβ, and aromatase activity) and hormone panel (e.g., LH, and FSH) measurements are missing. These require further molecular studies, which we are continuously conducting. Despite the shortcomings of our work, it is intended to raise awareness about the possible human hormonal effects of phytoecdysteroids. The human evidence for phytoecdysteroids is currently inconsistent. The variable and poor bioavailability, differences in metabolic pathways, and the unclear mechanism of action make it difficult to gain clarity about phytoecdysteroids, which is also reflected in our work. These clinical implications of the findings in humans require further research.

Moreover, a limitation of this study is the substantial number of parallel outcome measures analyzed across the six experimental groups (including body weight, multiple muscle types, and molecular/ELISA endpoints) without applying a global correction for multiple testing. While within-variable group comparisons were strictly controlled using Tukey’s post hoc test, we did not apply an omnibus correction for multiple comparisons across distinct outcome families. Because these physiological and molecular variables are biologically interconnected, strict alpha-adjustments could have overly suppressed statistical power. Nevertheless, readers should interpret these multiple parallel endpoints as an integrated pattern of biological shifts rather than completely independent statistical discoveries so that some findings may represent potential false positives.

## 6. Conclusions

In conclusion, CAL and 20E differ primarily in their sensitivity to reproductive and endocrine tissues, while they converge in their protection effects for metabolism and skeletal muscle. While CAL exhibits beneficial effects in weight regulation, anti-inflammatory activity, and muscle preservation, it also influences androgen receptor expression in the testicle. CAL may therefore possess unique advantages over 20E in preserving reproductive tissue integrity in obese rats, but the effect of increasing *Ar* and CYP19a1 mRNA expression in the ND group may be a cause for concern. Based on these findings in rats, we may have challenged the perception of phytoecdysteroids as completely harmless natural anabolic and underscore the need for caution in their use under certain conditions (depending on dose, diet, and developmental stage), the translation of which to humans requires further clinical studies.

The effects of CAL and 20E are likely due to tissue-specific receptor signaling and endocrine feedback complexity; however, future studies should explore dose–response relationships, explore long-term reproductive outcomes, include endocrine endpoints (e.g., LH/FSH profiling) and functional fertility outcomes (e.g., sperm quality and reproductive success) and explore tissue-specific receptor mechanisms to better define the safety and therapeutic potential of these compounds.

## Data Availability

All data utilized in this study can be found in the article. For additional information or requests regarding resources and reagents, please contact the lead investigator, Dr. Eszter Ducza, at ducza.eszter@szte.hu.
